# Cross-sectional study about primary health care professionals views on the inclusion of the vaccine against human papillomavirus in the vaccine schedules

**DOI:** 10.1186/s13027-015-0034-9

**Published:** 2015-11-16

**Authors:** M. Reyes Oliver Pérez, Victoria Bravo Violeta, Ana Vazquez del Campo, Cristina Ruiz, Sonia Yáñez Castaño, Laura P. Pérez Conde, Jesús S. Jiménez López

**Affiliations:** Department of Gynecologic Oncology, Hospital Universitario 12 de Octubre, Avda Cordoba s/n, Madrid, Spain; Department of Obstetrics and Gynecology, School of Medicine, Complutense University, Madrid, Spain

**Keywords:** Human papilloma virus, HPV vaccines, Cervical cancer, Primary care

## Abstract

**Background:**

Although the inclusion of the HPV vaccine has been registered in Spain since 2007, vaccination rates are lower than expected. The patients wish to be vaccinated is heavily influenced by information they have received from many source. The Knowledge of primary health care professionals affects the information provided to patients and is fundamental in the decision making. The aim of this study is to assess the opinions of primary health care professionals on the vaccine against HPV and their knowledge about HPV infection and its links to with gynecological and oropharyngeal cancer.

**Methods:**

Cross-sectional study. A 19-item survey was drawn up. It included questions on basic aspects of HPV infection and marketed vaccines, personal opinion about the inclusion in the immunization schedules and their level of prescription and recommendation to patients in their clinical practice. From October 2013 to December 2013, 607 surveys were distributed among 20 primary health centers affiliated to the University Hospital 12 de Octubre. The results were analyzed using SPSS statistical package.

**Results:**

One hundred sixty four successfully completed surveys were obtained for analysis. 89 % of the professionals knew about the relationship between HPV infection and cervical cancer, 57.3 % did not know any of the serotypes against which vaccines are targeted; 40.4 % believed that there is insufficient data to support the commercialization of the vaccines. Of these, 65.7 % argue that there is no data of its long-term effectiveness, 13.4 % that there is no data as to its side effects, 13.4 % believed that the cost effectiveness is not worthwhile.

**Conclusions:**

There is a strong controversy among health professionals regarding the marketing and inclusion of HPV vaccine in immunization schedules. However, the knowledge of the primary care health professionals on key aspects of infection and vaccine protection are insufficient. The training of professionals in vaccination, cervical pathology and HPV infection should be improved to provide objective information on the use as this vaccine for patients.

## Background

Human papilloma virus (HPV) infection is the most common sexual transmitted disease worldwide. The overall prevalence of the infection in Spain is 14.3 % (95 % Confidence interval (CI): 13.1 to 15.5), 28.8 % (95 % CI: 26.6 to 31, 1) in women between 18 and 25 [1]. HPV infection is considered the most important risk factor for developing cervical cancer [2]. The most frequently isolated genotypes in both malignant and premalignant disease are HPV 16 and 18, followed by 45 and 31 [3, 4].

Since 2006, two prophylactic HPV vaccines have been available in Spain: Gardasil® (Merck & Co., Whitehouse Station, NJ USA) and Cervarix® (GlaxoSmithKline Biologicals, Rixensart, Belgium). They consist of the native virus-like particles (VLPs), which are morphologically and immunogenetically similar to virions, but lack infectivity, replicative and tumorigenicity.

Both vaccines have demonstrated a high level of effectiveness against genotypes 16 and 18 [5, 6]. Cervarix® contains VLPs genotypes 16 and 18. It has been proved to be safe to administer in women up to 55 and has proved to generate protection for non-vaccine genotypes 31 and 45 [7]. Gardasil® contains VLPs genotypes 6, 11, 16 and 18. It has been also proven to be safe to administer in women up to 45 and in males.

United States, Australia, Canada and the UK were the first countries to introduce HPV vaccination into their routine immunization programs. In February 2007 Spain agreed to include the vaccine in the immunization schedules for girls between 11–14. Furthermore, it was agreed to recommend vaccination for all women up to age 26 and individualized assessment of vaccination in women over 26 years old and in males 9–26 years old out of public funding.

However, rates of vaccination in Spanish women have been lower than expected [8]. The controversy of the benefits of vaccination and its relation to possible adverse reactions, and the existence of groups of health care professionals with contrary opinions to the vaccine implementation, could play an important role in the low consumption of the vaccine. The more information we give to the patients, the higher the acceptance of the vaccine will be [9, 10]. The primary health care professionals (PHCP) are the first stage to transmiting information and opinion to patients.

The main objective of this study is to asses the opinions of PHCP of primary care centers affiliated to the Hospital 12 de Octubre (Madrid) on the vaccine against HPV and their knowledge about its characteristics and indications, and the basics concepts of HPV infection and its links to with gynecological and oropharyngeal cancers.

## Material and methods

Cross-sectional study. The research was conducted in 20 primary health centers attached to the Hospital 12 de Octubre.

**Fig. 1:**
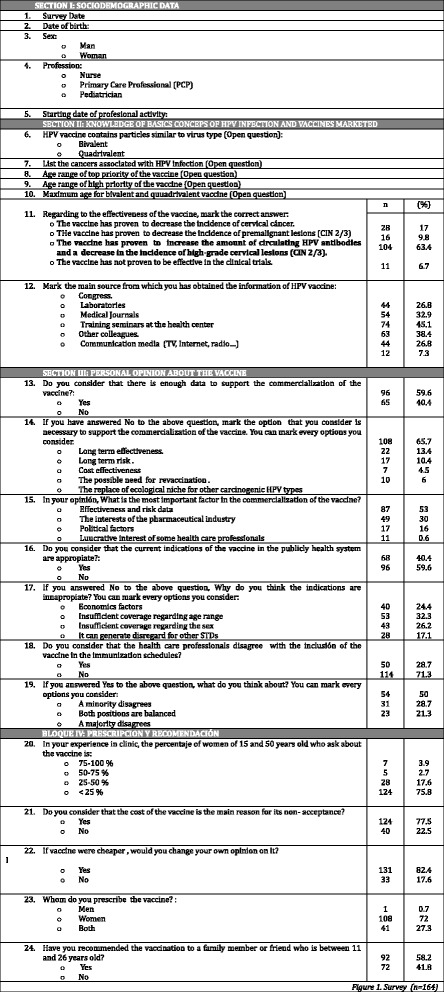
Survey

To obtain the data, we drew up a survey consisting of nineteen questions, 5 of open question format and 14 of multiple choice questions. The survey is divided in four sections (Fig. 1):

- Section I: Socio-demographic data, such as sex, age and occupation.
- Section II: Knowledge of basics concepts of HPV infection and vaccines marketed
- Section III: Personal opinion about the vaccine and its inclusion in the immunization schedules.
- Section IV: Prescribing and recommending the vaccine.

To design the survey, a literature review in databases the PubMed-Medline and UPTODATE was carried out. The keywords used were: Human papillomavirus, Vaccination, Cervical cancer.

The survey was aimed at PCHPs. Primary care physicians; primary care pediatricians and primary care nurses have been considered as PCHPs. Gynecologists have been excluded from the study because they are not the first stage to transmitting information and opinion on the primary prevention strategies to patients in our public health system.

The collected variables were included in an Excel database. Statistical analysis was performed with SPSS, version 15.0. Incomplete Surveys were not taken into account. We used the chi-square test to assess the significance of the association between two categorical variables. Statistical significance was considered as *p <* 0.05.

## Results

From October 2013 to December 2013, 607 surveys were delivered to 20 primary health centers attached to the Hospital 12 de Octubre; Of them, 171 were completed and 164 of these were valid for the analysis. The participation rate is 28 %. The percentage of men among those who replied was 25 and 65.2 % of women. The average age of participants was 46.6 years (SD ± 9.2, range 23–64) (Table 1).

**Table 1:** Socio-Demographic Data (*N* = 164)

Women	107 (65.2)
Men	57 (34.8)
Mean age (years)	46.6 ± 9.2 (23–64)
Proffesion :	
• PHCPs	97 (59.1)
• Nurse	51 (31.2)
• Pediatrician	16 (9.7)
Mean of years of professional experience (years)	18.8 ± 9.2 (1–39)
Value in n(%) or Mean ± ST (Range)	

### Section II: Knowledge of basics concepts of HPV infection and vaccines marketed

A total of 101 (61.6 %) of participants didn´t know any of the genotypes against which the bivalent vaccine (HPV 16 and 18) protects. The 60.4 % (99) of participants didn´t know any of the genotypes against which the quadrivalent vaccine protects (HPV 16,18,11,6). If we combine the above data, we find that 57.3 % (94) of those who replied didn´t know any of the genotypes against which both vaccines protect (Table 2).

**Table 2:** Knowledge of genotypes included in the vaccine (*N* = 164)

Bivalent vaccine (Cervarix ® contains VLPs of HPV 16 and 18)	
• Knowledge of HPV 16	1 (0.61)
• Knowledge of HPV 16 and 18	62 (37.8)
• No knowledge of any of them	101 (61.6)
Quadrivalent vaccine (Gardasil® contains VLPs OF HPV 16, 18, 11 y 6)	
• Knowledge of all of them	54 (32.9)
• No knowledge of any them	99(60.4)
• Knowledge of HPV 16 and 18	5 (3)
• Knowledge of HPV 11 and 18	1 (0.6)
• Knowledge of HPV 6 and 18	1 (0.6)
• Knowledge of HPV 6, 11 and 18	2 (1.22)
• Knowledge of HPV 11, 16 and 18	2 (1.22)
Global knowledge	
• Knowledge of every genotypes of both vaccine	46 (28)
• Knowledge of the genotypes of bivalent vaccine.	16 (9.7)
• Knowledge of the genotypes of quadrivalent vaccine.	8 (4.8)
• No knowledge of any of the serotypes	94 (57.3)
Value in n (%) or Mean ± ST (Range))	

Regarding the association between HPV infection and the development of cancer, 89 % (146) of those who replied knew the relationship between HPV and cervical cancer. However, as reflected in Table 3, between 68 and the 80 % of those who replied didn´t know that the relationship between HPV and the other carcinomas in which it´s involved.

**Table 3:** Comparison of professionals who support the vaccination vs. professionals who don´t support the vaccination

		Support the vaccination		
		Yes	No	*p*- value
Correct answer to question 11 (Effectiveness)	No	32 (45 %)	39 (54.9 %)	
	Yes	62 (72.9 %)	23 (27 %)	0.696
Information from congress	No	63 (53.4 %)	55 (46.6 %)	
	Yes	33 (76.8 %)	10 (23.2 %)	0.008
Information from training seminars	No	50 (51 %)	48 (49 %)	
	Yes	46 (73 %)	17 (27 %)	0.006
To consider appropriate the current indications for vaccination	No	50(52.6 %)	45 (47.4 %)	
	Yes	46 (71,8 %)	18 (28.2 %)	0.015

Regarding the indications of the vaccine, 7.9 % (13) answered that the highest priority age range of the vaccine is 9–14. A total of 20 (12.2 %) didn´t answer this question, and 80 % (130) of those who answered have done so incorrectly.

Referring to the effectiveness of the vaccine (question 11), 65.45 % (104) knew that the vaccine has proven to increase the amount of circulating antibodies and a decrease in the incidence of high-grade cervical lesions. However 17.6 % (28) wrongly answered that the vaccine has been demonstrated to decrease the incidence of Ca. Cervix.

Most of those who answered (45.1 %) reported having obtained the information in medical journals, followed by training seminars (38.41 %) and by pharmaceutical representatives (32.9 %).

### Section III: Personal opinion about the vaccine and its inclusion in the immunization schedules

The 71.3 % (114) of participants consider that there is no consensus of opinion on the inclusion of the vaccine in the calendar among health professionals. Of these, 50 % (54) considered that a majority agrees with its inclusion, 28.7 % (31) considered a balance between the two positions and 21.3 % (23) considered that a majority disagrees with its inclusion.

The 40.4 % (65) of those who answered think that there is insufficient data to support the commercialization of the vaccine. The most frequent reason given (65.7 %) is that they think there is no data on long-term effectiveness.

The 59.6 % (96) of the professionals vary on their opinion on the use of the vaccine in the public health system. The 32.3 % of those who answered think that the coverage of the age range is insufficient.

### Section IV: Prescribing and recommending the vaccine

According to the personal experience of the professionals, 75.8 % (124) of them, consider <25 % the percentage of women between 15 and 50 years old that ask for information about the vaccine, 17.6 % (28) consider this percentage to be around 25-50 %, 2.6 % (5) around 50-75 % and 3.9 % (7) in ≥75 %.

The 77.5 % (124) of participants thinks that the number of patients who agree to be vaccinated would be higher if it were cheaper. However, 82.4 % (131) did not change their own opinion despite decrease in price.

The 72 % (108) of participants recommended the vaccine only to women and 27.3 % (41) to both sexes; 59.8 % (98) recommended the vaccine to the age range of 14–26, 57.3 % (94) to the age range of 11–14 years, and 15.2 % (25) to the over 26. 58.2 % (92) of participants recommended vaccination of their own family members aged between 11 and 26.

When we analyzed the degree of recommendation of the vaccine based on proven knowledge about it (question 11) we notice that those professionals who correctly answered the question 11, recommend further vaccination (72.9 vs. 45 %; *p-value* 0.6). The belief that the vaccine has been shown to decrease the incidence of cervical cancer also implies an increase in the percentage of professionals who recommend the vaccine (23.4 vs. 9.7 %).

The recommendation of the vaccine is also influenced by the factors that could be used in marketing the professionals have considered to be the most influential. Those who think that the effectiveness and risk data are the most important factors, recommend the vaccine more (77.4 vs. 18.5 %), while those who think that the commercial interests of pharmaceutical industry is the most important factor, recommended the vaccine to a lesser degree (11.8 vs. 56.9 %).

Professionals who have obtained information from conference attendance recommend to a greater extent the routine vaccination against those who have not attended courses and conferences (76.8 vs. 53.4 %; *p-value* = 0.008). Likewise, those who have received the information from training seminars also recommend the routine vaccination more often (73 vs. 51 %; *p-value* 0.006).

## Discussion

The clinical trials published have demonstrated an effectiveness of nearly 100 % of both vaccines in women between 15 and 26 years old. Also, high immunogenicity in children under 15 years old has been demonstrated [11, 12]. This has conditioned the modification of vaccination schedules in many countries.

Although the risk of HPV infection is higher at the beginning of sexual activity, it remains high throughout the sex life. Each year, 5-15 % of middle-aged women acquire a new infection [13, 14]. On the other hand, the older the patient, the higher the probability of persistent infection and, therefore, increased risk of development cervical cancer. Several studies have confirmed the effectiveness of the quadrivalent vaccine in women until the age of 45 and the bivalent vaccine in women older than 26 [15, 16].

In Spain, the systematic vaccination against HPV was established in February 2007 for children between 11–14 years. Out of public funding, it was agreed to recommend vaccination for all women younger than 26 years and individualized assessment of vaccination to women over 26 years and in males 9–26 years. However, rates of vaccination in the population included in the public funding are lower than expected [8].

The vaccination rates are directly associated with the information provided to women on HPV infection and the characteristics of the vaccines [9]. The PHCPs are the first stage transmitting information and recommending the vaccine. The information given by professionals to the patients is conditioned by their own knowledge of the vaccines and their personal opinion on them.

In 2008, the Spanish scientific societies published a consensus document, based on the available evidence of the HPV vaccine, establishing the indications for the use of the vaccine. Three years later, in 2011, the impact of that document hadn´t been as successful as it was to thought it should have been. Because of persistent doubts about the usefulness of the recommendations on vaccination against HPV, its effectiveness and risk [17] a new consensus document focused on HPV vaccination was published in 2011 [18].

In our study, we analyzed the knowledge of PCPs about HPV infection and the basic characteristics of the vaccines. The 89.2 % of participants know the relationship between HPV and cervical cancer. However, it highlights their lack of Knowledge about genotypes included in the vaccines (57.3 % didn´t know the serotypes against which both vaccines protect), and the relationship of HPV with cancer of the vagina, penis, vulva, oropharynx or anus (79.88, 73.78, 71.95, 70.12 and 68.29 %).

One of the most important data obtained in our study is that 40.4 % of participants think that there is insufficient data to support the commercialization of the vaccine. The main reason they consider (65.7 %) is there are no long-term studies on the influence of vaccination on mortality from cervical cancer. The 13.4 % state that there are no studies of long-term risk of the vaccine, cost effectiveness (10.4 %), the possible need for revaccination (4.5 %) and the replacement of ecological niche for other HPV types (6 %).

Recently, Naud et al. have published their data of a long-term study with 9.4 years of follow-up in women vaccinated with Cervarix®. The effectiveness against CIN 2 was 100 % and 100 % of women remained seropositive against serotypes 16 and 18, with titers at least 10 times higher compared with natural immunity [19]. In addition, there are mathematical models that predict that the antibodies are to remain high for at least 20 years or even a lifetime [20].

Approximately 120 million doses of vaccine have been distributed worldwide. No serious adverse effects related to the vaccine have been observed in none of the clinical trials done [16, 18, 21]. The most frequently reported adverse effects were transient and at the part of the body where it was injected [22].

Regarding the cost effectiveness of the introduction of the vaccine as part of routine vaccination, the World Health Organization has established that HPV vaccination combined with a redesigned screening is the most effective strategy for the prevention of cervical cancer [23]. Castellsagué et al. studied the impact of the vaccine in Spain with a mathematical model simulation. According to their conclusion with the combination of systematic vaccination in children, the stimulation of vaccination in young women outside public funding and the current screening programs, 83.5 % of total costs associated with these diseases (168 million euros per year) would be reduced [24, 25].

On the other hand, note the perception of 75.8 % of participants that less than 25 % of women between 15 and 50 ask for the vaccine. This may be due to of a lack of information or knowledge within the general population about the implications of HPV infection, its causal association with cervical cancer and the importance of primary prevention. One of the causes for low immunization coverage in young adult women described in several epidemiological studies is the lack of awareness about the disease and concern about side effects of vaccination [26].

The main factor associated with the use of vaccine is the information that women have about the mechanism of HPV infection and its implication in several cancers and the effects of vaccination in terms of effectiveness and risk. It is important emphasize on the advantages of the systematic vaccination and individualization of each case in women over 14 [26].

The PHCPs are the first patient contact with the programs of primary and secondary prevention. The information that these professionals provide to their patients influences their decision-making. Therefore, such information must be based on the available scientific evidence.

Our study shows that there is a knowledge gap in primary health care professionals on basic aspects of HPV infection and its associated lesions, as well as the characteristics of vaccines and the population susceptible to receive it.

It is essential to ensure the training and continuous updating of professionals to provide dissemination of appropriate information to our patients and, consequently, facilitate and improve national HPV vaccine coverage.

## Conclusions

The information given to patients about HPV, cancer and vaccine are the most important factors on vaccination (greater knowledge, greater acceptance). The knowledge and training of primary care professionals in this area should be enhanced, in order to improve the information given to our patients.

### Limitations of study

Our results cannot be extrapolated to all PHCPs in Spain. Although the participation rate is acceptable and similar to those obtained by other authors such as Torné et al. [26] with a share of 40.4 %, or Mazzadi et al. [27] with a share of 15.23 %, it still remain a low percentage. It could be biased towards those professionals most motivated in participate.

## Abbreviations

HPV: Human papilloma virus
PCPs: Primary care professionals
STD: Sexual transmitted disease
CI: Confidence interval
VLPs: Native virus-like particles
GP: General practitioners
CIN: Cervical intraepithelial neoplasia
WHO: World Health Organization
